# New Insights on Using Oral Semaglutide versus Dapagliflozin in Patients with Type 2 Diabetes and Metabolic Dysfunction-Associated Steatotic Liver Disease

**DOI:** 10.3390/diagnostics14141475

**Published:** 2024-07-10

**Authors:** Ermina Stratina, Carol Stanciu, Robert Nastasa, Sebastian Zenovia, Remus Stafie, Adrian Rotaru, Tudor Cuciureanu, Cristina Muzica, Catalin Sfarti, Irina Girleanu, Horia Minea, Oana Petrea, Laura Huiban, Stefan Chiriac, Ana-Maria Singeap, Oana Vlad, Camelia Cojocariu, Anca Trifan

**Affiliations:** 1 Department of Gastroenterology, Grigore T. Popa University of Medicine and Pharmacy, 700115 Iasi, Romania; stratina.ermina@yahoo.com (E.S.); stanciucarol@yahoo.com (C.S.); sebastianzenovia20@gmail.com (S.Z.); stafieremus@gmail.com (R.S.); adrianrotaru94@yahoo.com (A.R.); drcuciureanutudor@gmail.com (T.C.); lungu.christina@yahoo.com (C.M.); cvsfarti@gmail.com (C.S.); gilda_iri25@yahoo.com (I.G.); horia.minea@yahoo.com (H.M.); stoica_oanacristina@yahoo.com (O.P.); laura.huiban@yahoo.com (L.H.); stefannchiriac@yahoo.com (S.C.); anamaria.singeap@yahoo.com (A.-M.S.); cameliacojocariu@yahoo.com (C.C.); ancatrifan@yahoo.com (A.T.); 2 “St. Spiridon” Emergency Hospital, Institute of Gastroenterology and Hepatology, 700111 Iasi, Romania; 3 Unit of Diabetes, Nutrition and Metabolic Diseases, Grigore T. Popa University of Medicine and Pharmacy, 700115 Iasi, Romania; oana_vlad1981@yahoo.com

**Keywords:** type 2 diabetes mellitus, GLP1-analogs, SGLT2-inhibitors, liver fibrosis, MASLD

## Abstract

Background and aims: Increases in both the prevalence and severity of metabolic dysfunction-associated steatotic liver disease (MASLD) and obesity are closely related. Type 2 diabetes (T2DM) has been associated with metabolic dysfunction-associated steatohepatitis (MASH)-related cirrhosis and hepatocellular carcinoma. Semaglutide is a glucagon-like peptide-1 (GLP-1) receptor agonist approved for the treatment of T2DM and has an important role in weight loss. Also, it may represent a new therapeutic option for the treatment of MASH in obese diabetic patients. The main outcomes were changes from baseline in liver steatosis and fibrosis at week 24. Material and methods: A total of one hundred eighty-seven patients with T2DM were eligible for this prospective study; ninety-five subjects were treated with oral semaglutide, and ninety-two patients were treated with dapagliflozin as an add-on to metformin. All the subjects were evaluated using Vibration Controlled Transient Elastography (VCTE) from June to December 2022. Results: From our cohort, 54% of the patients were females, with a mean age of 59.92 ± 11.89 years and a mean body mass index (BMI) of 29.53 ± 5.33 kg/m^2^. Following a six-month medication period, we observed a substantial reduction in anthropometric measurements, including the BMI, waist circumference (WC), and waist-to-hip ratio (WtHr), in both groups. Regarding HbA1c, a notable decrease was observed in the semaglutide group (*p* < 0.001) when compared to the dapagliflozin group (*p* = 0.011). In addition, the liver stiffness measurement (LSM) according to VCTE improved significantly in the semaglutide group participants from 8.07 ± 2.90 kPa at baseline to 6.51 ± 3.09 kPa after medication (*p* < 0.001). Conclusion: The superior metabolic effects of semaglutide, correlated to dapagliflozin, may contribute to a more efficient decrease in hepatic stress and injury, leading to a substantial enhancement of liver function in T2DM patients. Further investigations conducted over an ideal timeframe are necessary to confirm the evidence presented in this study.

## 1. Introduction

The strong connection between insulin resistance and non-alcoholic fatty liver disease (NAFLD) is also reflected in the most recent nomenclature updates for liver steatosis, in which NAFLD was initially renamed metabolic dysfunction-associated fatty liver disease (MAFLD) and subsequently metabolic dysfunction-associated steatotic liver disease (MASLD). MASLD is diagnosed in the presence of radiological signs of steatosis in individuals with obesity or diabetes mellitus, as per these definitions. In the case of lean individuals, the diagnosis requires two metabolic risk abnormalities, one of which is prediabetes [1].

Glucagon-like peptide-1 (GLP-1) receptor agonists (GLP-1RAs) have been widely recognized as a viable therapeutic approach for managing type 2 diabetes (T2DM). They have been specifically designed to stimulate the receptor of the hormone GLP-1, a significant incretin hormone that stimulates the human body’s glucoregulatory response. It is released by nutrient-responsive enteroendocrine cells or L cells of the intestine. GLP-1 enhances the release of insulin, which is dependent on glucose, optimizes the function of β-cells and glucose tolerance, blocks the release of glucagon, and provides defense against apoptosis and glucolipotoxicity [2]. Through an intrinsic gut–liver signaling route, GLP-1 is essential in reducing insulin resistance and in very low-density lipoprotein synthesis [3]. The presence of GLP-1 receptors in hepatocytes has been a topic of discussion among researchers. GLP-1 stimulates the production of cyclic adenosine monophosphate (cAMP) in liver cells, leading to the activation of cAMP-activated protein kinase (AMPK), which decreases the production of lipids. Preclinical research involving individuals with metabolic dysfunction-associated steatohepatitis (MASH) indicated that GLP-1 RAs may decrease hepatic inflammation through processes that are partially unrelated to weight loss. From a mechanistic standpoint, it is important to note that hepatocytes, stellate cells, and Kupffer cells do not possess the classical GLP-1 receptors. Therefore, the effects of GLP-1 on the liver are mostly mediated by indirect pathways [4,5]. Moreover, GLP-1 plays a significant part in the process of digestion through its ability to inhibit gastric emptying and suppress the appetite center located in the hypothalamus [6]. 

Individuals diagnosed with MASH face a significantly elevated susceptibility to the development of severe liver-related complications, including portal hypertension, hepatic decompensation, hepatocellular carcinoma, liver-related mortality, and cardiovascular events, which could represent a threat to their lives [7].

Type 2 diabetes affects 71% of patients with MASH-related cirrhosis, and inadequate glycemic control is associated with advanced disease and poor clinical outcomes. As there are currently no approved medications for the treatment of MASH, these patients have a great unmet need for effective pharmacotherapies to improve the natural history of this disease, including the associated increased risk for cardiovascular morbidity and mortality [8]. 

GLP-1RAs have demonstrated the ability to induce effects across multiple organs. In individuals with T2DM, GLP-1RAs have been observed to decrease glycated hemoglobin (HbA1c) levels while also promoting weight reduction in those who are overweight or obese [9]. Furthermore, GLP-1RAs have been linked to a diminished likelihood of experiencing adverse cardiovascular events in patients with T2DM who possess a heightened risk of cardiovascular complications [10]. In a prior placebo-controlled experiment, it was observed that the GLP-1RA semaglutide demonstrated improvements in metabolic indices and remission of MASH in patients without cirrhosis. Additionally, the treatment was well tolerated by the patients [11].

Newsome et al. recently published a phase 2 trial of subcutaneous semaglutide in patients with biopsy-proven MASH and utilized a parallel-group design. This study was randomized, double-blind, placebo-controlled, and multicentered. The trial included 320 patients and showed that the proportion of patients who have improved the histological aspect of MASH with no progression of fibrosis was significantly greater in every semaglutide group [12].

It is well known that glucose-regulating sodium–glucose co-transporter type-2 (SGLT-2) inhibitors (SGLT-2i) decrease serum uric acid levels, with weight loss being achieved concurrently [13]. Approval for use in non-diabetic patients with heart failure and chronic kidney disease has been granted to these agents due to the enormous benefits they have demonstrated even in the absence of diabetes [14]. SGLT-2i possesses robust antioxidant and anti-inflammatory properties, rendering it as a potentially efficacious therapy option for MASH in addition to its anti-hyperglycemic and weight reduction effects [15].

Due to the improvement of biological markers of MASLD and imaging techniques, recent data from animal studies and clinical trials have shown that SGLT-2i reduces fatty liver accumulation. However, these benefits have been observed primarily in patients with T2DM [16].

This study’s objective was to assess the semaglutide’s effectiveness, safety, and tolerability versus dapagliflozin as an add-on to metformin in patients with T2DM and obesity.

## 2. Materials and Methods

### 2.1. Patients

**Figure 1:**
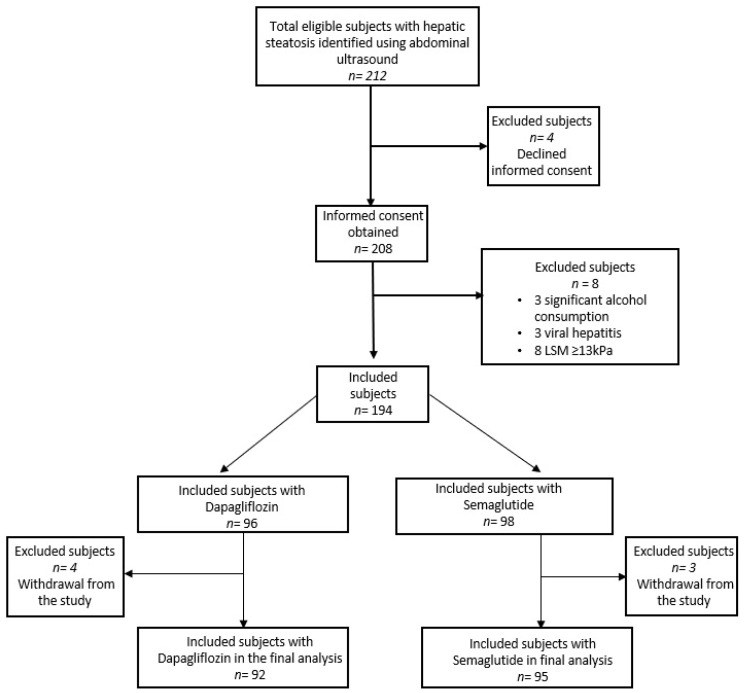
Study participant flow chart.

A total of 212 patients with T2DM were eligible for this prospective study, but according to Figure 1, only 187 individuals were recruited in the final analysis. The participants were drawn from the Saint Spiridon Emergency Hospital’s Gastroenterology and Diabetes Department. Individuals who met the following requirements and were over the age of 18 were considered for the study: they had to be able to give informed consent; they had to have T2DM, either with or without prescription medication; their HbA1c level had to be more than 6.5% (48 mmol/mol); they had to have a controlled attenuation parameter (CAP value ≥ 274 dB/m using transient elastography) to show evidence of hepatic steatosis; and they were going to start SGLT-2i or GLP-1 analog therapy. Internal medicine and/or diabetology board-licensed physicians made the selection of certain medications and oversaw each patient’s course of care. The following were some of the therapy plans: regarding GLP-1 analogs, start with a daily dose of 3 mg of oral semaglutide and increase to 7 mg after 30 days. Only 17 individuals needed further glycemic reduction, and the dose was increased to 14 mg once daily, which was reduced to 7 mg after one month. As an alternative, individuals with SGLT-2i therapy used a dose of 10 mg of dapagliflozin once daily. 

The following were among the exclusion criteria: the concomitance of liver cirrhosis (liver stiffness measurement (LSM) during elastography ≥ 13 kPa) or the hepatitis B/C/D or other causes of chronic liver diseases (autoimmune hepatitis, Wilson’s disease, hemochromatosis, primary biliary cholangitis, right-sided heart failure, HIV co-infection, alcoholic liver disease; alcohol consumption ≥ 21 drinks/ week (30 g alcohol/d) in men and ≥14 drinks/week (20 g alcohol/d); acute coronary syndrome, stroke, or transient ischemic attack in the last 3 months before informed consent; impaired kidney function (estimated glomerular filtration rate < 30 mL/min/1.73 m^2^) during screening; contraindications to metformin according to the local label; medical history of cancer or treatment for cancer within the last 5 years; treatment with systemic steroids at the time of consent; change in the dosage of thyroid hormones within 6 weeks before consent. Also, individuals with unreliable or failed VCTE measurements were excluded from this analysis. 

The Ethics Committee of our Hospital endorsed the study, which was conducted in compliance with the Declaration of Helsinki’s tenets. Before the assessment, each subject gave their informed consent.

### 2.2. Study Procedures and Clinical Examination

On the same day, each patient underwent a thorough clinical examination, lab tests, and a VCTE evaluation. Information on gender, age, regular alcohol and tobacco use, body mass index (BMI), type of diabetic medication, and systolic and diastolic blood pressure were among the demographic and clinical data collected. Patients who met the study requirements underwent a baseline examination, and they were monitored for up to six months after starting SGLT-2i or GLP-1 analogs. Medication modifications made during the study or withdrawal of the patients were recorded, as was adherence to the previously prescribed drugs using the pill-count (or comparable) technique.

### 2.3. Biological Tests and VCTE Examinations

Hemoglobin, the international normalized ratio (INR), fibrinogen, fasting plasma glucose, HbA1c, albumin, total proteins, urea, serum creatinine, triglycerides, high and low-density lipoprotein, bilirubin, and serum uric acid were among the blood indicators that were measured. The presence of chronic viral hepatitis (HBV/HCV or HBV + HDV) was identified using HBs Ag, anti-HCV, and anti-HVD antibodies.

Before VCTE exams, a qualified physician who specialized in liver imaging performed an abdominal ultrasound scan using a 3.5–5 MHz convex probe and a high-resolution B-mode scanner (Supersonic Aixplorer^®^ MACH 30, Supersonic Imagine, Aix-en-Provence, France). The study assessed the presence and intensity of fatty liver by examining four ultrasonographic indicators: hepatorenal contrast, bright liver, deep attenuation, and vessel blurring. The US fatty liver scoring system was used to categorize participants into three groups based on the grade of fatty liver: non-fatty liver group (0 points), mild fatty liver group (1–3 points), and fatty liver group (4–6 points) [17]. Subsequent, patients were evaluated for liver fibrosis and steatosis using the FibroScan^®^ 520 Compact model (Echosens, Paris, France) equipped with the M- (normal) or XL- (obese) probe by a single operator with over 300 determinations in VCTE practice. After at least four hours of fasting, patients were placed in the supine position with the right arm at maximal abduction. As a result, the intercostal window for liver scanning of the right lobe was expanded. An automatic machine indication signaled the use of the XL-probe (2.5 MHz) when the distance between the skin and the liver capsule exceeded 25 mm. Initially, the M-probe with a 3.5 MHz transducer frequency was used for the test. A measurement was considered accurate if ten acquisitions were made with an interquartile range divided by the median (IQR/M) of no more than 30%. The LSM and CAP measurements were performed at baseline and 24 weeks. According to CAP measurement, a quantitative technique expressed in decibel-milliwatts (dB/m) ranging from 100 to 400 dB/m, the cut-off values for mild (S1), moderate (S2), and severe steatosis (S3) were, respectively, ≥274 dB/m, ≥290 dB/m, and ≥302 dB/m [18]. The cut-off values for LSM examinations were as follows: 5.6 kPa for mild fibrosis (F1), 8 kPa for significant fibrosis (F2), 9.6 kPa for advanced fibrosis (F3), and 13 kPa for cirrhosis (F4) [19].

### 2.4. Non-Invasive Tests for the Evaluation of Liver Fibrosis

In addition, we computed the FIB-4 index (which includes age, aspartate aminotransferase (AST), alanine aminotransferase (ALT), and platelets) for each patient, the NFS score (which includes age, BMI, AST, ALT, albumin, platelets, and T2DM status), and the AST-to-platelet ratio index (APRI). These tests performed better in head-to-head comparisons than other straightforward non-invasive fibrosis tests, especially in MASLD subjects. Additionally, the originally reported formula was used to calculate the AGILE 3+ score, which considers age, sex, AST, ALT, platelets, T2DM status, and LSM. Patients were categorized as having a low risk of advanced fibrosis if their score was less than 0.45, an intermediate risk between 0.45 and 0.67, and a high risk greater than 0.68. Additionally, to determine their alcohol use, everyone filled out the AUDIT-C questionnaire. According to current scientific recommendations, the threshold that governs persons with excessive alcohol intake is typically 20 g per day for women and 30 g per day for men [20].

### 2.5. Anthropometric of Body Composition

Anthropometric measurements were performed using a weight scale and a height meter, and measures of each patient enrolled in the study were taken. The World Health Organization established the cut-off values for lean (≥18.5 kg/m^2^), overweight (≥25 kg/m^2^), and obese (>30 kg/m^2^) individuals. Additionally, the waist-to-height ratio (WtHR), which divides waist circumference (in centimeters) by height (in centimeters), was computed as an alternative indicator of obesity and settled at ≥0.50. Abdominal obesity was defined as a waist circumference of ≥80 cm in women and ≥94 cm in men, which is also thought to be an approximation for visceral adiposity [21].

### 2.6. Study Outcomes and Statistics

The primary outcome was changes in hepatic steatosis and fibrosis using VCTE with CAP after six months of therapy with GLP-1 Ras or SGLT-2i. Changes in body composition, liver function tests (LFTs), lipid status, glucose metabolism indicators, and liver fibrosis non-invasive tests (Fib-4 index, NFS-score, APRI, and Agiles 3+ score) were among the secondary outcomes. Subgroup analysis investigated the impacts of the two kinds of drugs (SGLT-2i and GLP-1 analogs). The normality of the data distributions was tested using the Kolmogorov–Smirnov and Shapiro–Wilk tests. The results were reported using the mean ± standard deviation or the median (interquartile range), depending on the test results. The Wilcoxon signed rank test and the Student’s *t*-test were used to examine differences between two dependent samples, while the Mann-Whitney U test and the *t*-test were used to assess independent samples. For related samples, changes across time were evaluated using the Friedman test and repeated measures ANOVA. The median (interquartile range) is used to represent the data for the subgroup analysis if the normal distribution assumption is significantly rejected. Absolute changes in CAP at six months served as the dependent variable in a post hoc linear regression, whereas the following covariates served as independent variables: baseline CAP, baseline BMI, baseline glucose, and baseline weight changes. GraphPad Prism 7.0 (GraphPad Software) and SPSS 20.0 (IBM) were used for the analyses. Statistical significance was evaluated by a two-sided *p*-value ≤ 0.05.

## 3. Results

### 3.1. Patient Characteristics

In the final analysis, we included 187 individuals who had T2DM and hepatic steatosis according to a CAP value ≥ 274 dB/m. From this cohort, 54% were females, with a mean age of 59.92 ± 11.89 years and a mean BMI of 29.53 ± 5.33 kg/m^2^. Also, our study population had important comorbidities such as hypertension, which was found in 70.1% of the subjects, and the presence of metabolic syndrome was also described in 81.2% of the individuals. After 6 months of therapy, we observed a significant decrease in anthropometric parameters such as the BMI, WC, and WtHr in both groups, with a higher decrease in patients that take semaglutide (*p* < 0.001) in comparison with the dapagliflozin group (*p* = 0.005). In terms of biological parameters, we observed a slight improvement of creatinine in both groups with no significant differences between the semaglutide group (*p* = 0.038) and the dapagliflozin group (*p* = 0.034). Regarding liver function tests, we observed a better improvement of ALT (from 57.18 ± 26.78 IU/L to 33.18 ± 12.8 IU/L) and AST (from 55.8 ± 25.32 to 35.28 ± 13.65) in semaglutide individuals, but with a significant statistical *p*-value in both groups (*p* < 0.001). 

**Figure 2:**
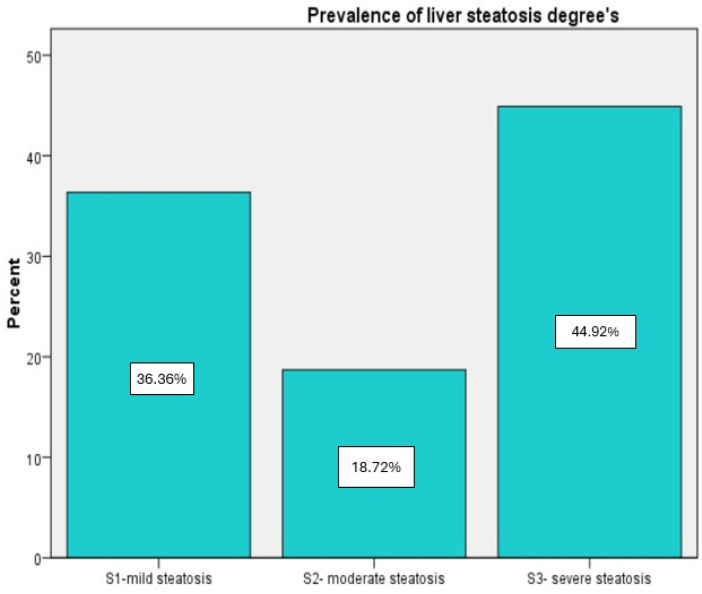
Prevalence of liver steatosis.

**Figure 3:**
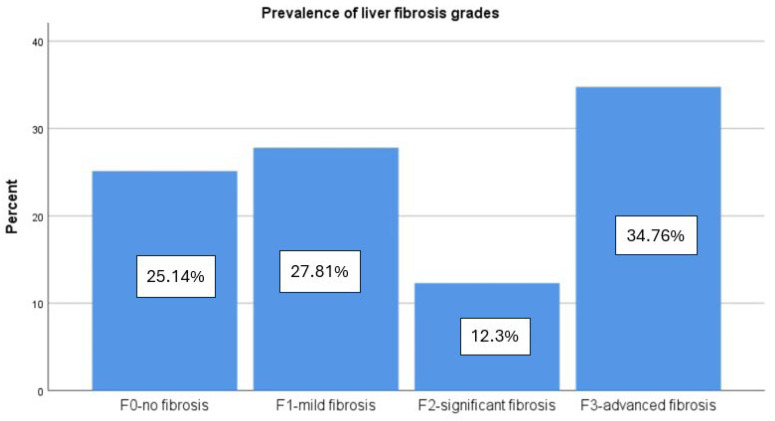
Prevalence of liver fibrosis grades.

According to VCTE measurements, the mean CAP value was 311.57 ± 35.59 dB/m, which indicates severe hepatic fat accumulation. Most of the individuals (44.92%) had severe steatosis degree (S3), the moderate form (S2) was described in 35 (18.72%) patients, and the mild steatosis (S1) affected more than one-third of the subjects (36.36%) (Figure 2). Additionally, LSM presents a mean value of 8.12 ± 2.99 kPa, which indicates histologically significant liver fibrosis grade (F2). Therefore, 47 (25.13%) of the individuals had no fibrosis, 52 (27.81%) had mild fibrosis, 23 (12.3%) had significant fibrosis, and 65 (34.67%) had advanced fibrosis as specified by VCTE evaluation (Figure 3). All the non-invasive fibrosis scores present a significant decrease after 6 months of medication with a *p* < 0.001, which was also described for LSM values with a mean of 7.27 ± 2.17 kPa (*p* = 0.011). Additionally, these diminished levels were found in the CAP score too, with a mean value of 293.28 ± 33.65 dB/m (*p* < 0.001) after antidiabetic treatment (Table 1).

**Figure 4:**
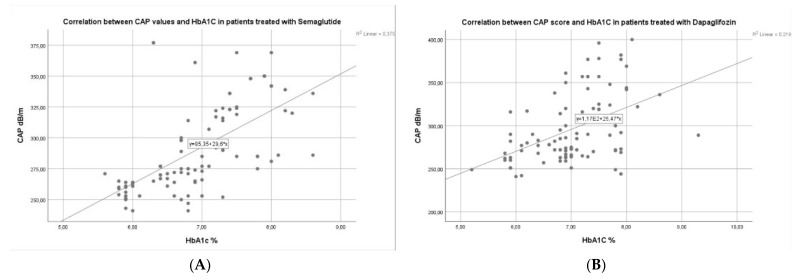
(**A**,**B**) Correlation between CAP values and HbA1c in semaglutide and dapagliflozin group.

The metabolically biological parameters such as fasting glucose and HbA1c were markedly diminished after 6 months of each of the therapies, with a significant reduction in the semaglutide group of HbA1c (*p* < 0.001) in comparison to dapagliflozin individuals (*p* = 0.011) (Table 1). Additionally, we found a significantly higher correlation between CAP values and HbA1c after 6 months of semaglutide therapy (r^2^ = 0.375), than in subjects who took dapagliflozin for 6 months (r^2^ = 0.219) (Figure 4A,B). Also, in patients that take analogs of GLP-1, we observed a significant reduction of TG from 189.14 ± 58.46 mg/dL at the baseline to 175.89 ± 56.32 mg/dL (*p* = 0.032) at 6 months, in comparison with subjects that takes SGLT2 inhibitors who do not present a significant reduction in TG (p= 0.062). Concerning non-invasive biomarkers of liver fibrosis, we observed a remarkable reduction in NFS score (*p* = 0.029), Fib-4 index (*p* = 0.004), APRI score (*p* < 0.001), and Agile3+ score (*p* < 0.001) in semaglutide group which was not found in patients that takes dapagliflozin (*p* = 0.061) (Table 1).

**Figure 5:**
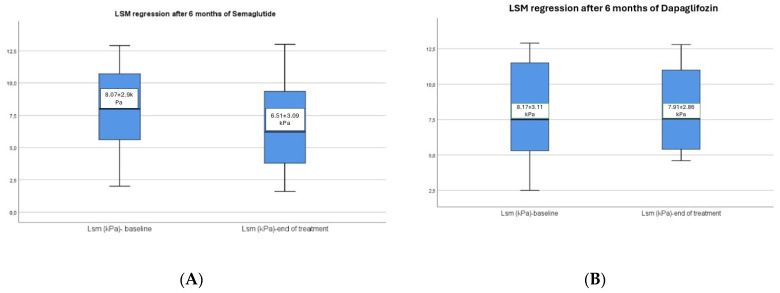
(**A**,**B**) LSM regression after 6 months of semaglutide vs. dapagliflozin.

**Figure 6:**
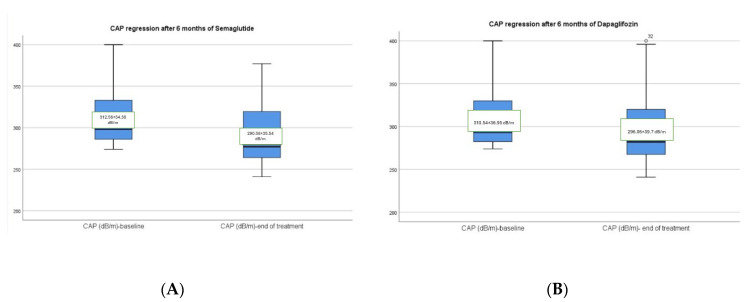
(**A**,**B**) CAP regression after 6 months of semaglutide vs. dapagliflozin.

Moreover, participants who took semaglutide had a significant improvement of LSM according to VCTE from 8.07 ± 2.90 kPa at the baseline to 6.51 ± 3.09 kPa after therapy (*p* < 0.001) in comparison with dapagliflozin group with a slight reduction of LSM values from 8.17 ± 3.11 kPa to 7.91 ± 2.86 kPa, but without significant difference (*p* = 0.074) (Figure 5A,B). Another significant improvement in the semaglutide group was the decrease of CAP values from 312.56 ± 34.38 dB/m to 290.58 ± 35.54 dB/m (*p* < 0.001) in contrast with the dapagliflozin group which presents a significant reduction of the CAP values from 310.54 ± 36.95 dB/m to 296.06 ± 39.7 dB/m with a statistical *p*-value of 0.014, which indicates a lower level of confidence (Figure 6A,B).

### 3.2. Hepatic Changes Associated with Semaglutide and Dapagliflozin Treatment

**Figure 7:**
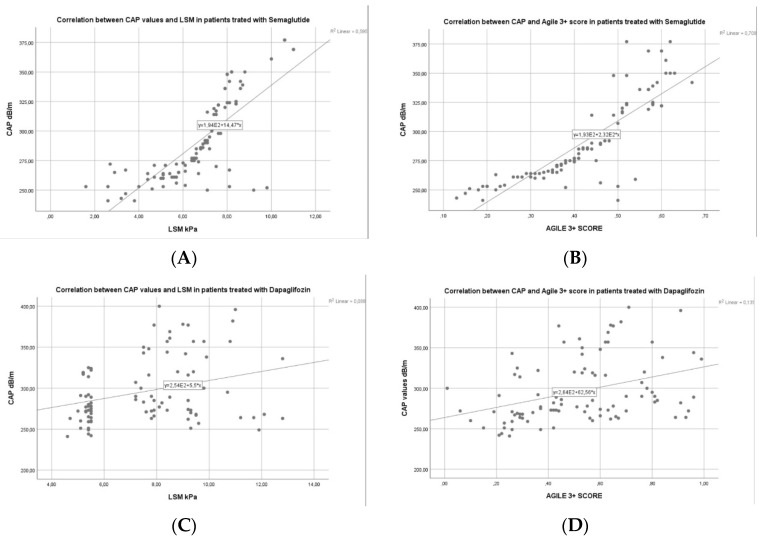
(**A**–**D**) Correlation between CAP and LSM or Agile3+ score in both groups.

**Figure 8:**
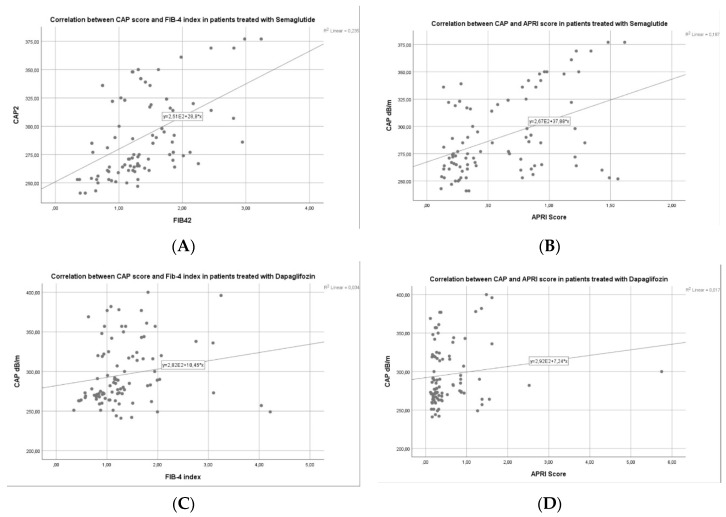
(**A**–**D**) Correlation between CAP values and non-invasive biomarkers of liver fibrosis in both groups.

Approximately 2 out of 5 individuals (41.71%) who take semaglutide or dapagliflozin therapy presented with no steatosis (S0) after the regimen, 32.62% were found with mild steatosis (S1), 8.56% subjects had moderate steatosis (S2), and 17.11% had severe steatosis (S3) according to CAP values obtained by VCTE after treatment. Also, there was a significant improvement in the liver fibrosis grades after therapy as follows: 46.52% had no fibrosis (F0), 16.04% had mild fibrosis (F1), 13.9% had significant fibrosis (F2), and 23.53% of the individuals had advanced fibrosis (F3). Moreover, we found an important correlation between CAP and LSM (r^2^ = 0.595) and CAP and Agile-3+ score (r^2^ = 0.701) in the semaglutide group in comparison with dapagliflozin group with a mild correlation with a r^2^ = 0.088, and r^2^ = 0.135, respectively (Figure 7A–D). Additionally, we found a mild correlation between CAP and FIB-4 index (r^2^ = 0.235), and CAP and APRI score (r^2^ = 0.187) in the GLP-1 group in comparison with SGLT-2 inhibitors, where we found no statistical correlation between CAP and FIB-4 index (r^2^ = 0.034) and CAP and APRI score (r^2^ = 0.017) (Figure 8A–D).

### 3.3. Factors Associated with Liver Steatosis Response for Patients with Semaglutide vs. Dapagliflozin

We performed a univariate linear regression analysis to establish risk factors associated with liver steatosis response (defined by decreased CAP values after 6 months of therapy) comparing both groups of patients (semaglutide vs. dapagliflozin). Only, those factors which had a significant *p*-value (*p* < 0.05) were included in the multivariate regression analysis (Table 2). In univariate analysis, we observed that in the semaglutide group, the BMI at baseline (β = 0.496, *p* < 0.001), WC at baseline (β = 0.356, *p* = 0.018), WtHr at baseline (β = 0.254, *p* = 0.012), CAP at baseline (β = −0.474, *p* = 0.004), glucose at baseline (β = 0.321, *p* = 0.012), HbA1c at baseline (β = 0.298, *p* = 0.007), and TG at baseline (β = 0.277, *p* = 0.006) were significant factors of absolute CAP modification after 6 months of therapy. Instead, in the dapagliflozin group, we noticed that the BMI at baseline (β = 0.423, *p* = 0.066), WC at baseline (β = 0.322, *p* = 0.034), WtHr (β = 0.241, *p* = 0.022), CAP at baseline (β = −0.438, *p* = 0.008), glucose at baseline (β = 0.315, *p* = 0.013) and HbA1c at baseline (β = 0.272, *p* = 0.021) were the factors associated with CAP changes after SGLT-2i treatment, but no influence of TG baseline levels in this group. In multivariate analysis, we found that the BMI at baseline (β = 0.491, *p* = 0.009), CAP at baseline (β = −0.468, *p* = 0.014), glucose at baseline (β = 0.313, *p* = 0.028), HbA1c (β = 0.292, *p* = 0.016), and TG at baseline (β = 0.269, *p* = 0.015) were independent predictors of CAP modification after semaglutide therapy. On the other hand, in the dapagliflozin group, only the BMI at baseline (β = 0.415, *p* = 0.017) and glucose at baseline (β = 0.306, *p* = 0.031) were independent factors associated with CAP changes after treatment with SGLT-2 inhibitor drugs (Table 2).

## 4. Discussion

Modern health systems are particularly burdened by MASLD, which impacts a substantial number of individuals and is accompanied by major mortality and morbidity. In support of the so-called “multiple parallel hit” model, genetic and epigenetic factors have been implicated in the pathogenesis of the disease. In this hypothesis, multiple “hits” interact dynamically to propel the diagnosis and progression of MASLD. Various substances and therapies have been attempted to treat this pathology, with mixed results [22]. A recent meta-analysis by Younossi et al. finds that MASLD is 55% prevalent worldwide among patients with T2DM, indicating that it is a common condition among this population [23]. Given the critical role that insulin resistance plays in the development of MASLD, it is only inevitable that antidiabetic medications require extensive research on these patients [24]. In our study, we assessed the outcomes of treatment with SGLT2i and GLP1 RAs in MASLD individuals with T2DM. 

Following a six-month treatment period, a substantial reduction in anthropometric parameters, including the BMI, WC, and WtHr, was observed in both groups. Notably, patients undergoing semaglutide treatment experienced a more pronounced decrease in all these elements (*p* < 0.001) compared to those in the dapagliflozin group (*p* = 0.005). Also, metabolically derived parameters, including fasting glucose and HbA1c, decreased significantly, and semaglutide patients reported the most pronounced decrease. Our results are supported by Ding et al., who showed in a recent meta-analysis that both SGLT-2i and GLP-1 analogs cause weight loss and improve glycemic control, highlighting a greater efficacy of semaglutide compared to SGLT-2i [25]. In the SUSTAIN 8 study, semaglutide showed a substantially larger decrease in HbA1c levels compared to the SGLT-2i Canagliflozin after 1 year. The mean reduction in HbA1c levels was 1.5% for semaglutide and 1.0% for Canagliflozin [26]. After six months, participants in our trial who were given GLP-1 analogs showed a significant reduction in CAP (312.56 ± 34.38 dB/m to 290.58 ± 35.54 dB/m). This is in line with recent research that shows GLP-1 analogs significantly improve hepatic steatosis. Arai et al. found that 24-week oral semaglutide treatment in patients with MASLD complicated by T2DM impaired liver function and liver steatosis (CAP values decreased from 344 dB/m to 279 dB/m) [27].

Regarding effects on composite indices of hepatic steatosis and fibrosis, NFS score (*p* = 0.029), FIB-4 index (*p* = 0.004), APRI score (*p* < 0.001), and Agile3+ score (*p* < 0.001) were significantly reduced in the semaglutide group, a finding not observed in the dapagliflozin patient group. The improving values of AST and ALT levels may account for the decrease in the FIB-4 index values and might signify an amelioration in liver fibrosis [28]. Extrahepatic complications, including cardiovascular events and non-liver malignancies, account for a significant proportion of fatalities and are correlated with the FIB-4 index. A prognosis improvement and prevention of these complications may result from GLP-1 Ras treatment in patients with MASLD [29]. Consistent with these findings, Gameil et al. examine the impact of Liraglutide and Dulaglutide with standard treatment on fatty liver index (FLI) and FIB-4 index in patients with T2DM and MASLD. The administration of liraglutide and dulaglutide resulted in notable enhancements in the FIB-4 measurements, with a more substantial modification found in the liraglutide cohort [30]. In addition, participants who received semaglutide saw a notable enhancement in LSM as determined by VCTE. The LSM decreased from 8.07 ± 2.90 kPa at the beginning of the study to 6.51 ± 3.09 kPa after the therapy (*p* < 0.001), which was a substantial improvement compared to the group that received dapagliflozin. In contrast, Flint et al. discovered that semaglutide can lead to a reduction of over 30% in hepatic steatosis compared to a placebo. However, there was no significant improvement observed in liver stiffness as assessed by magnetic resonance elastography (MRE) or in the magnetic resonance imaging-estimated proton density fat fraction (MRI-PDFF) [31]. Although there was no disparity in the enhancement of fibrosis, Newsome’s study revealed that a lesser percentage of patients in the semaglutide group (5%) exhibited a worsening of fibrosis compared to the control group (19%), highlighting an eventual advantage [32].

In the group of patients who were treated with semaglutide, a statistically significant decrease in ALT (from 57.18 ± 26.78 IU/L to 33.18 ± 12.8 IU/L) and AST (from 55.8 ± 25.32 to 35.28 ± 13.65) values was observed compared to patients who received dapagliflozin. Kai Zhu et al. assessed the effect of semaglutide on ALT and AST and demonstrated notable decreases of 14.06 U/L and 11.44 U/L, respectively, in comparison to the placebo [33]. However, Kumar et al. conducted a comprehensive analysis of 26 trials and determined that GLP-1 Ras is linked to a noteworthy decrease in ALT and GGT levels while having no statistically significant impact on AST values. They also exhibited a substantial decrease in hepatic steatosis, with inadequate data regarding an effect on inflammation and fibrosis [34].

The findings of a post hoc univariate linear regression analysis indicate that baseline BMI, WC, WtHr, CAP, glucose, HbA1c, and TG were significant drivers of CAP improvement in the semaglutide group. On the other hand, in the dapagliflozin group, we noticed that the BMI, CAP, fasting plasma glucose, and HbA1c at baseline were the factors associated with CAP changes after SGLT-2i treatment. After that, multivariate regression analysis indicated that the BMI, CAP, glucose, HbA1c, and TG at baseline were the parameters associated with CAP improvement in the semaglutide group. The only independent parameters linked with changes in CAP after treatment with SGLT-2i medications were the baseline BMI and baseline glucose levels. Our findings are supported by Mittag-Roussou et al., whose study included 39 diabetic patients treated with GLP-1 analogs and SGLT-2 inhibitors, having as factors associated with the improvement of hepatic steatosis BMI, CAP, and fasting plasma glucose [35]. The number of participants, the study design, and the pertinent head-to-head comparison with a well-established glucose-lowering medication are all strengths of our study. 

The current study does have certain limitations. First, considering that measuring the effect of key potential confounding factors, such as calorie consumption and physical exercise, was not possible. Second, the trial was conducted on patients with a wide range of LSM from low to extremely high, and the period of treatment was limited in investigating the effect of semaglutide and dapagliflozin on liver fibrosis. Third, several patients in the study had very high LSM or severe obesity, and LSM and CAP were probably not adequately evaluated in these circumstances with the FibroScan 502. Therefore, the evaluation by liver biopsy would have been necessary for a more accurate evaluation of the impact of the treatments on liver function.

## 5. Conclusions

In conclusion, in patients with uncontrolled T2DM and MASLD/MASH, oral semaglutide treatment was superior to dapagliflozin added to metformin in reducing HbA1c, body weight, TG, and LDLc. The two treatments received favorable responses, with minimal occurrences of hypoglycemia. These findings contribute to the existing body of evidence from the SUSTAIN clinical trial program, demonstrating that semaglutide is an efficient medicine for reducing glucose levels. It also provides supplementary advantages, including weight loss and protective effects on the liver [25]. Therefore, future studies conducted over an optimal period are needed to validate the data presented in this research and to accurately evaluate the impact of GLP-1 analogs on liver function in patients with T2DM and MASLD.

## Figures and Tables

**Table 1:** Comparison of subgroup patients treated with semaglutide or dapagliflozin.

	Semaglutide Group			Dapagliflozin Group		
	Baseline (*n* = 95)	6 mo (*n* = 95)	*p*	Baseline (*n* = 92)	6 mo (*n* = 92)	*p*
Sex F, *n* (%)	52 (54.7)			50 (54.3)		
Age (y)	56 ± 9.85			61.47 ± 11.22		
Hypertension, *n* (%)	67 (70.5)			64 (69.6)		
Dyslipidemia, *n* (%)	53 (55.8)			47 (51.1)		
Metabolic syndrome, *n* (%)	79 (83.15)			73 (79.3)		
Smoking status, *n* (%)	24 (25.3)			29 (31.5)		
BMI (kg/m^2^)	29.49 ± 5.21	27.32 ± 4.61	<0.001	29.57 ± 5.47	27.86 ± 4.85	0.005
Waist circumference (cm)	98.34 ± 9.8	94.8 ± 8.91	<0.001	97.72 ± 9.66	95.13 ± 4.42	0.008
WtHr	0.57 ± 0.07	0.51 ± 0.04	<0.001	0.59 ± 0.09	0.54 ± 0.04	0.007
Normal weight, *n* (%)	17 (17.9)	24 (25.3)	0.016	16 (17.4)	24 (26.1)	0.009
Overweight, *n* (%)	32 (33.7)	42 (44.2)	0.006	30 (32.6)	33 (35.8)	0.072
Obesity, *n* (%)	46 (48.4)	29 (30.5)	<0.001	46 (50)	35 (38.1)	0.011
Biological parameters						
HGB (g/dL)	13.48 ± 1.69	13.27 ± 1.62	0.452	13.27 ± 1.67	13.06 ± 1.63	0.106
Platelet count (g/L)	216.01 ± 65.48	218.95 ± 67.01	0.236	225.68 ± 69.09	228.98 ± 71.22	0.291
CRP (mg/dL)	0.72 ± 0.31	0.7 ± 0.29	0.185	0.76 ± 0.33	0.73 ± 0.28	0.162
Ferritin (ng/mL)	149.63 ± 57.94	144.01 ± 56.2	0.264	137.16 ± 54.82	131.08 ± 52.16	0.314
Bilirubin (mg/dL)	0.82 ± 0.44	0.84 ± 0.45	0.322	0.88 ± 0.47	0.85 ± 0.43	0.106
Total proteins (g/dL)	7.63 ± 0.69	7.85 ± 0.72	0.081	7.63 ± 0.68	7.83 ± 0.71	0.161
Albumin (g/dL)	4.73 ± 0.55	4.79 ± 0.63	0.076	4.71 ± 0.56	4.77 ± 0.6	0.320
BUN (mg/dL)	40.94 ± 23.26	37.03 ± 22.84	0.075	38.32 ± 20.01	37.75 ± 19.66	0.370
Creatinine (mg/dL) Serum uric acid (mg/dL)	0.83 ± 0.3 6.46 ± 0.75	0.72 ± 0.27 6.41 ± 0.73	0.038 0.102	0.81 ± 0.26 6.59 ± 0.8	0.71 ± 0.24 6.48 ± 0.76	0.034 0.088
ALT (IU/L) AST (IU/L)	57.18 ± 26.78 55.8 ± 25.32	33.18 ± 12.8 35.28 ± 13.65	<0.001 <0.001	54.06 ± 24.82 56.7 ±26.1	38.16 ± 14.6 39.11 ±15.2	<0.001 <0.001
Fasting glucose (mg/dL)	147.46 ± 41.07	112.52 ± 36.23	<0.001	137.96 ± 40.78	108.44 ± 33.81	<0.001
HbA1c (%)	8.11 ± 0.84	6.94 ± 0.74	<0.001	7.75 ± 0.81	7.01 ± 0.72	0.010
Cholesterol (mg/dL) Triglycerides (mg/dL)	221.24 ± 60.96 189.14 ± 58.46	219.69 ± 60.02 175.89 ± 56.32	0.143 0.032	211.38 ± 57.51 174.7 ± 56.24	208.96 ± 56.68 168.54 ± 55.36	0.211 0.062
LDL-C (mg/dL)	154.29 ± 46.7	142.26 ± 43.38	0.027	141.57 ± 41.96	139.95 ± 41.02	0.195
HDL-C (mg/dL)	45.55 ± 9.53	47.38 ± 9.71	0.090	45.29 ± 9.46	47.11 ± 9.84	0.093
LF non-invasive markers						
FIB-4 index	1.79 ± 1.12	1.36 ± 0.89	0.004	1.62 ± 0.95	1.34 ± 0.79	0.036
NFS-score APRI score Agile 3+ score	0.54 ± 1.01 0.58 ± 0.2 0.48 ± 0.18	0.42 ± 0.96 0.46 ± 0.17 0.38 ± 0.12	0.029 <0.001 <0.001	0.52 ± 1.04 0.56 ± 0.21 0.44 ± 0.16	0.46 ± 0.97 0.5 ± 0.21 0.4 ± 0.15	0.046 0.037 0.061

BMI, body mass index; WtHr, waist to height ratio; HGB, hemoglobin; CRP, c-reactive protein; ALT, alanine aminotransferase; AST, aspartate aminotransferase; BUN, blood urea nitrogen; HbA1C, Glycated hemoglobin; LDL-C, low-density lipoprotein cholesterol; HDL-C, high-density lipoprotein cholesterol; LF, liver fibrosis; FIB-4, fibrosis-4 index; NFS, NAFLD fibrosis score; APRI, aspartate aminotransferase to platelet ratio index.

**Table 2:** Univariate and multivariate linear regression analysis of factors associated with decreased CAP values after treatment with semaglutide vs. dapagliflozin.

	Semaglutide Group				Dapagliflozin Group			
	Univariate		Multivariate		Univariate		Multivariate	
Parameter	Β	*p*	Β	*p*	β	*p*	β	*p*
BMI at baseline	0.496	<0.001	0.491	0.009	0.423	0.006	0.415	0.017
WC at baseline	0.356	0.008	0.347	0.023	0.322	0.034	0.318	0.079
WtHr at baseline	0.254	0.012	0.248	0.047	0.241	0.022	0.239	0.094
CAP at baseline	0.474	0.004	−0.468	0.014	−0.438	0.008	−0.0429	0.022
Glucose at baseline	0.321	0.012	0.313	0.028	0.315	0.013	0.306	0.031
HbA1c at baseline	0.298	0.007	0.292	0.016	0.272	0.021	0.269	0.054
TG at baseline	0.277	0.006	0.269	0.015	0.268	0.088	-	-

## Data Availability

The data presented in this study are available on request from the corresponding author. The data are not publicly available because they are the property of the Institute of Gastroenterology and Hepatology, Iasi, Romania.
